# Supplementary material for: The genome sequence of a soldier beetle, Malthinus seriepunctatus Kiesenwetter, 1851
Source: Wellcome Open Res. 2025 Feb 19;10:80. [Version 1] doi: 10.12688/wellcomeopenres.23716.1 (PMC11955078; doi:10.12688/wellcomeopenres.23716.1)
Supplement: Peer review history

# Reviewer report: Reviewer response for version 1

**DOI:** 10.21956/wellcomeopenres.26164.r121191

**Published:** 2025-04-04

**Authors:** Doga Cedden

**Affiliations:** 1 University of Göttingen, Göttingen, Germany

Mitchell et al. report a high quality and chromosomally complete genome assembly from a soldier beetle, *Malthinus seriepunctatus. *The background information is sufficient and methodology is adequately described. Authors present sufficient quality control of the genome assembly, which is publicly available. 

 Comments:

 1) I suggest putting a scale bar in Figure 1.

 2) Antennae are not visible in Figure 1. Were they cut by the authors prior to DNA extraction? If so, this could be mentioned in the Methods.

Are sufficient details of methods and materials provided to allow replication by others?

Yes

Is the rationale for creating the dataset(s) clearly described?

Yes

Are the datasets clearly presented in a useable and accessible format?

Yes

Are the protocols appropriate and is the work technically sound?

Yes

Reviewer Expertise:

I work on coleopteran pests using RNA-seq and RNA interference.

I confirm that I have read this submission and believe that I have an appropriate level of expertise to confirm that it is of an acceptable scientific standard.

# Reviewer report: Reviewer response for version 1

**DOI:** 10.21956/wellcomeopenres.26164.r121201

**Published:** 2025-03-29

**Authors:** Hume Douglas

**Affiliations:** 1 Agriculture and Agri-Food Canada, Ottawa, Canada

The article is generally well written.

**I have just two questions:**

- What was the source of your diagnostic statement please? Did this come from Duff, Wittmer, or your own observations?
- What literature or specimen sources did M. Geiser use to identify the specimen?

This will be a useful contribution to phylogenetic progress toward a good classification of the Elateroidea

Are sufficient details of methods and materials provided to allow replication by others?

Partly

Is the rationale for creating the dataset(s) clearly described?

Yes

Are the datasets clearly presented in a useable and accessible format?

Yes

Are the protocols appropriate and is the work technically sound?

Yes

Reviewer Expertise:

Beetle taxonomy including phylogenetics

I confirm that I have read this submission and believe that I have an appropriate level of expertise to confirm that it is of an acceptable scientific standard.

# Reviewer report: Reviewer response for version 1

**DOI:** 10.21956/wellcomeopenres.26164.r119263

**Published:** 2025-03-11

**Authors:** Zachary P Cohen

**Affiliations:** 1 United States Department of Agriculture, College Station, USA

This report represents the highest degree of thoroughness, relevance and readability. The near-chromosomal quality genome of this soldier beetle, is the first of its kind for this specious genera, *Malthinus*, but follows existing high quality genome assemblies of soldier beetle by the consortium. I appreciate this group's prolific contributions; however, if feasible, I would encourage referencing existing WGS of other *Cantharidae* spp, perhaps in the abstract, to provide additional context for the reader. Here, like with other DTLC reports, the team effectively describes genomic features and data quality as well as the relevant biological and ecological contexts for this species. The materials and methods are easy to follow and reproduce; the figures, images & tables are clear and legible. The datasets are clearly presented.

Are sufficient details of methods and materials provided to allow replication by others?

Yes

Is the rationale for creating the dataset(s) clearly described?

Yes

Are the datasets clearly presented in a useable and accessible format?

Yes

Are the protocols appropriate and is the work technically sound?

Yes

Reviewer Expertise:

Insect genomics and evolution.

I confirm that I have read this submission and believe that I have an appropriate level of expertise to confirm that it is of an acceptable scientific standard.
